# Pollen atlas and identification dataset from Santa Catarina, south Brazil

**DOI:** 10.1016/j.dib.2022.108855

**Published:** 2022-12-25

**Authors:** Erika Rodrigues, Marcelo Cancela Lisboa Cohen, Beatriz L Figueiredo, Kam-biu Liu, Qiang Yao

**Affiliations:** a Department of Oceanography and Coastal Sciences and Coastal Studies Institute, Louisiana State University, Baton Rouge, LA 70803, United States; b Institute of Geosciences, University of São Paulo, São Paulo, Brazil; c Graduate Program of Geology and Geochemistry, Federal University of Pará, Av. Perimentral 2651, Terra Firme, 66077-530 Belém, PA, Brazil; d University of São Paulo, CENA/^14^C Laboratory, Av. Centenário 303, 13400-000 Piracicaba, São Paulo, Brazil

**Keywords:** Palynology, Paleoecology, Microfossil, Paleontology

## Abstract

Pollen references such as atlas and identification keys are remarkably rare from the Atlantic coasts of south Brazil. This dataset describes modern and fossil pollen from São Francisco do Sul Bay in the state of Santa Catarina, south Brazil. An illustrated and descriptive atlas of pollen was compiled from original and published data to facilitate the identification of various pollen taxa in sediments. We include light micrographs and descriptions of a total of 60 pollen taxa, including 3 mangrove taxa, 27 coastal woodland (restinga) taxa, and 15 rainforest taxa, and 15 herbaceous taxa. This dataset is associated with a published research article by Rodrigues et al. (2022) - Rodrigues, E., Cohen, M.C.L., Pessenda, L.C.R., França, M.C., Magalhães, E. and Yao, Q., 2022. Poleward mangrove expansion in South America coincides with MCA and CWP: A diatom, pollen, and organic geochemistry study. Quaternary Science Reviews, 288, p.107598.

**Specifications table**

**Table:** 

Subject	Earth and Planetary Sciences - Paleontology
Specific subject area	Pollen atlas and identification Key
Type of data	Image and table
How the data were acquired	The images were acquired via an Olympus Light Microscope with a LC35 camera (3.5-megapixel CMOS sensor) in conjunction with ZEN Blue 2012 imaging software to photograph pollen grains under 1000x magnification.
Data format	Raw
Description of data collection	Pollen identification was based on microstructural analysis of the aperture and ornamentation, such as the characteristics of pore, colpus, and texture. At least 300 pollen grains were counted for each sample.
Data source location	• City/Town/Region: São Francisco do Sul Bay, Santa Catarina, south Brazil • Country: Brazil • Latitude and longitude (and GPS coordinates, if possible) for collected samples/data: 26°06′48.10′′ S, 48°47′17.00′′ W
Data accessibility	Repository name: Mendeley Data Data identification number: DOI: 10.17632/hr2z5yxtnw.2 Direct URL to data: https://data.mendeley.com/datasets/hr2z5yxtnw
Related research article	Rodrigues, E., Cohen, M.C.L., Pessenda, L.C.R., França, M.C., Magalhães, E. and Yao, Q., 2022. Poleward mangrove expansion in South America coincides with MCA and CWP: A diatom, pollen, and organic geochemistry study. Quaternary Science Reviews, 288, p.107598. https://doi.org/10.1016/j.quascirev.2022.107598

**Value of the Data**

• This pollen atlas facilitates the identification of microfossil pollen from mangrove, coastal woodland, Atlantic rainforest, and dune environments.
• The relatively abundance of different pollen taxa permits the assessment of vegetation, morphology, salinity, and other environmental factors in the modern- and paleo-ecological research.
• The concentration of pollen reveals the vegetation dynamics and productivity in the Atlantic coast of south Brazil from the Holocene to the Anthropocene.

## 1 Objective

Although the millennial scale vegetation dynamics is revealed by microfossil analysis in the published research article by Rodrigues et al. [1], pollen atlas and identification key cannot be incorporated due to the scope of the study. This article aims to provide an illustrated and descriptive pollen atlas from the Atlantic coast of south Brazil. Such illustrative dataset will provide a useful reference to researchers from across the globe to duplicate our findings from south Brazil and serve as a baseline data for future studies to reveal the vegetation dynamics in a millennial timescale and document the eco**-**morphological evolution of the coastal zone along the Atlantic coast of south Brazil.

## 2 Data Description

Microfossil pollen analysis is one of the most widely used proxy in the reconstruction of paleoenvironmental change and vegetation dynamics worldwide. Microfossil pollen analysis provides a unique perspective to reveal the temperature, morphology, and other environmental parameters in a decadal to millennial timescale.

However, pollen atlas and identification key are remarkable rare from the Atlantic coast of south Brazil, particularly from the state of Santa Catarina, resulting an obstacle in the palynological analysis of the region. This dataset provides an illustrated and descriptive atlas that describes modern and fossil pollen from a 100 cm sediment core retrieved from an estuarine mangrove swamp adjacent to the upstream of Palmital River's main channel, near São Francisco do Sul Bay, south Brazil. Currently, vegetation along the Atlantic coast of south Brazil exhibits a zonation pattern consisting of four different units: mangrove, restinga (coastal woodland), the Atlantic rainforest (montana dense ombrophilous and lowland ombrophilous forest), and dune (herbaceous) vegetation [1]. This dataset aims to facilitate the identification of various pollen taxa in sediments. We include light micrographs and detailed descriptions of a total of 60 pollen taxa, including 3 mangrove taxa, 27 coastal woodland (restinga) taxa, 15 rainforest taxa, and 15 herbaceous taxa. The original count and relative abundance of all the pollen taxa is listed in Mendeley Data [2]. These data can be used as a reference for future studies to conduct pollen analysis in south Brazil.

**Fig. 1:**
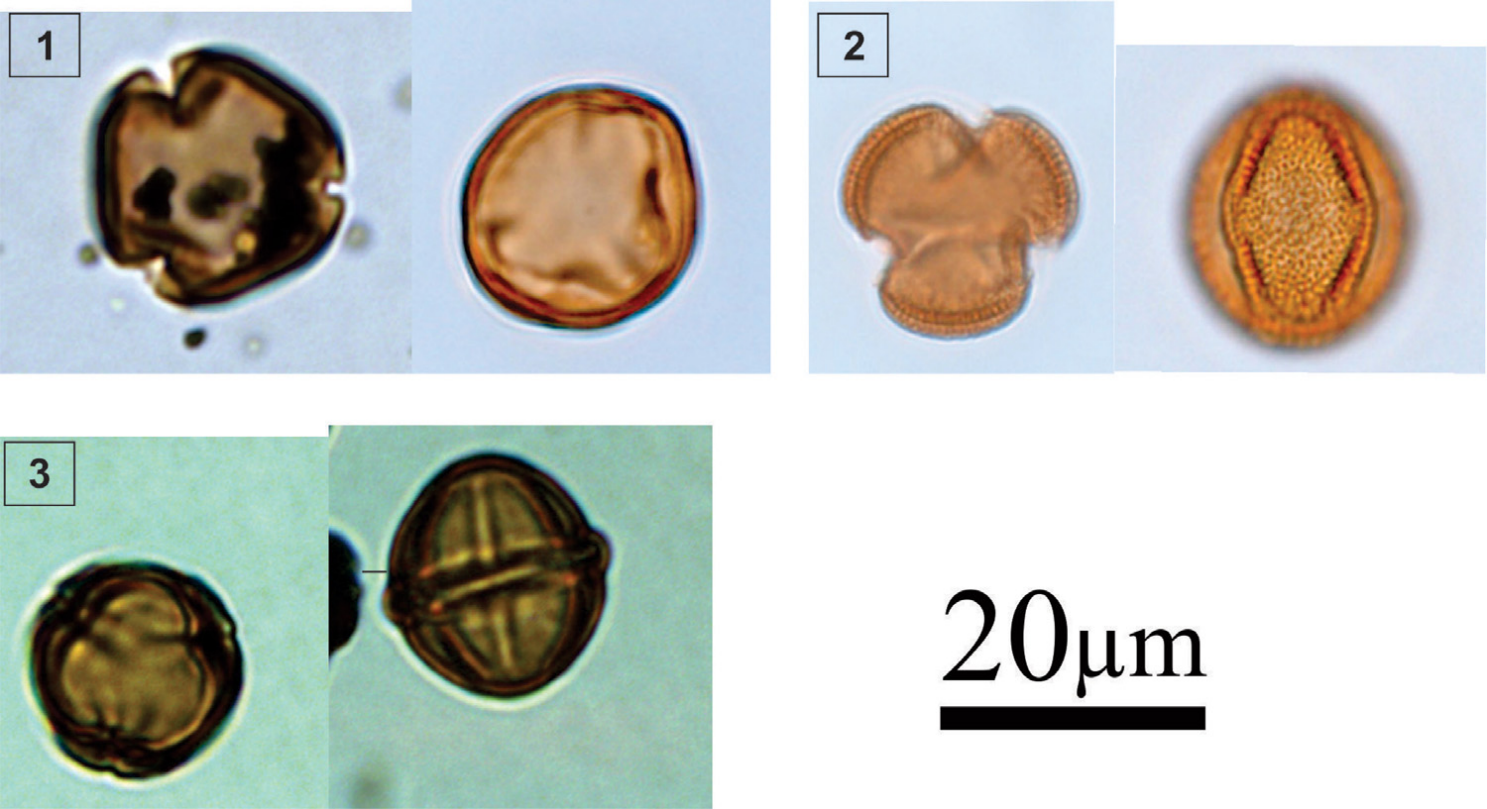
Light micrographs of mangrove taxa, including: **1.***Laguncularia racemosa* (Combretaceae); **2.***Avicennia germinans* (Acanthaceae); **3.***Rhizophora mangle* (Rhizophoraceae).

### 2.1 Identification key (1-3; Fig. 1)

1. ***Laguncularia racemosa* (Combretaceae; white mangrove) [** **2** **]**

Subspheroidal to prolate grain; tricolporate; finely reticulate; colpus length: 17–22 µm; oval pore; polar axis: 22–28 µm; equatorial axis: 15–23 µm

2. ***Avicennia germinans* (Acanthaceae; black mangrove)**

Spheroidal grain, tricolporate; reticulate; colpus length: 20–25 µm; elongated pore; polar axis: 32–38 µm; equatorial axis: 25–30 µm

3. ***Rhizophora mangle* (Rhizophoraceae; red mangrove)**

Subspheroidal to prolate grain; tricolporate; finely reticulate; colpus length: 15–20 µm; lalongate pore; polar axis: 22–27 µm; equatorial axis: 17–21 µm

**Fig. 2:**
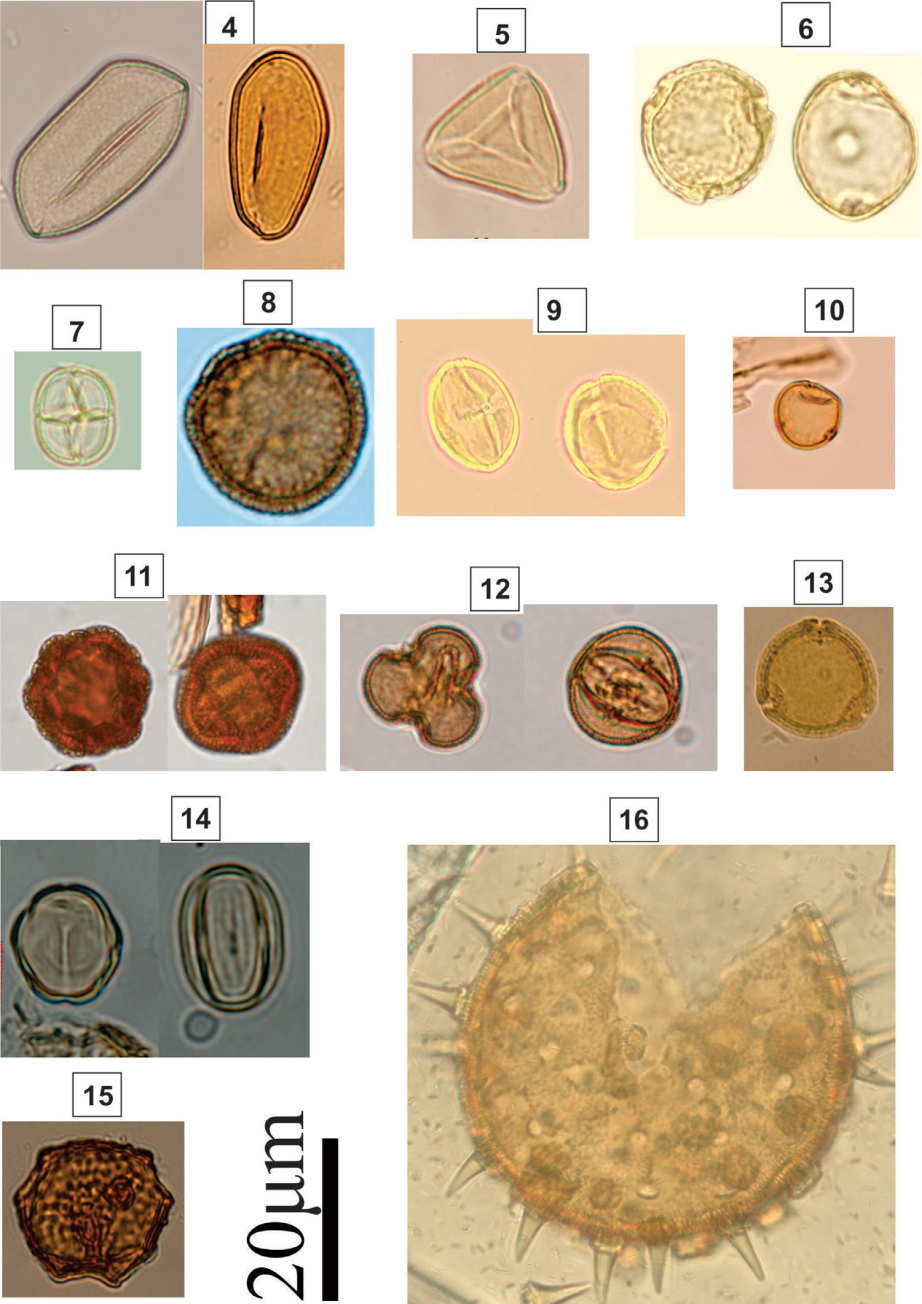
Light micrographs of coastal woodland (restinga) taxa, including: **4.***Butia* sp*.* (Arecaceae); **5.***Matayba* sp. (Sapindaceae); **6.***Symplocos* sp.1 (Symplocaceae); **7.***Mimosa* sp. (Mimosoidae); **8.***Hedyosmum* sp. (Chloranthaceae); **9.***Tapirira* sp. (Anacardiaceae); **10.***Celtis* sp. (Ulmaceae); **11.***Borreria* sp. (Rubiaceae); **12.***Sebastiania* sp. (Euphorbiaceae); **13.***Symplocos* sp. 2 (Symplocaceae); **14.***Miconia* sp*.* (Melastomataceae); **15.***Erythrina* sp. (Fabaceae); **16.***Hibiscus* sp. (Malvaceae)

### 2.2 Identification Key (4-16; Fig. 2)

4. **Arecaceae**

Monosulcate, pitted to micropitted.

5. ***Matayba* sp. (Sapindaceae)**

Triangulate grains; tricolporate; syncopated; straight to concave triangular amb; oblate; microreticulate; circular pores; medium size; Polar axis (14.5–15.7 µm); Equatorial axis (26.7–31.1 µm).

6. ***Symplocos* sp.1 (Symplocaceae)**

Spherical grain; tricolporate; exine tectate 2 µm thick; sexine verrucate; pores apparently circular, polar area as wide as grain; circular amb.

7. ***Mimosa* sp. (Mimosoidae)**

Uniplanar tetragonal tetrad; psilate; size: 11 µm; Shrub.

8. ***Hedyosmum* sp. (Chloranthaceae)**

Spherical grain; apolar; asymmetric; inaperturate; sexine clavate; densely scattered on the surface; amb circular; grain spheroidal; size: 38 µm.

9. ***Tapirira* sp. (Anacardiaceae)**

Subspheroidal grain; Tricolporate; striated; subprolate; colpus length: 18–24 µm; lalongate pore; polar axis: 18–24 µm; equatorial axis: 18–21 µm

10. ***Celtis* sp. (Ulmaceae)**

Spherical grain; diporate; microrugulate; round pore; size: 18.0–21

11. ***Borreria* sp. (Rubiaceae)**

Subspheroidal grain; heterocolporate; reticulate; colpus length: 19–25 µm; round annulate pore; polar axis: 20–23 µm; equatorial axis: 18–28 µm

12. ***Sebastiana* sp. (Euphorbiaceae)**

Subspheroidal to prolate; tricolporate; isopolar; microreticulated; colpus width: 0.5–3 µm; circular to elliptical pore; polar axis: 23–30 µm; equatorial axis: 19–24 µm

13. ***Symplocos* sp. 2 (Symplocaceae)**

Spherical grain; tricolporate; short colpus; convex triangular amb; psilate; Exine thick in pores forming oculus.

14. ***Miconia* sp*.* (Melastomataceae)**

Oval grain; isopolar; symmetric; heterocolpate; sexine psilate; pores inconspicuous; hexalobed amb; subprolate; small pollen; polar axis: 8 µm; equatorial axis: 15 µm.

15. ***Erythrina* sp. (Fabaceae)**

Subspheroidal grain; triporate; suboblate; reticulate; circular pore; homobrocates; polar axis: 28, 9–29, 5 µm; equatorial axis: 32, 2–33, 8 µm.

16. ***Hibiscus* sp. (Malvaceae)**

Spherical grain; periporate; echinate; size: 30–50 µm

**Fig. 3:**
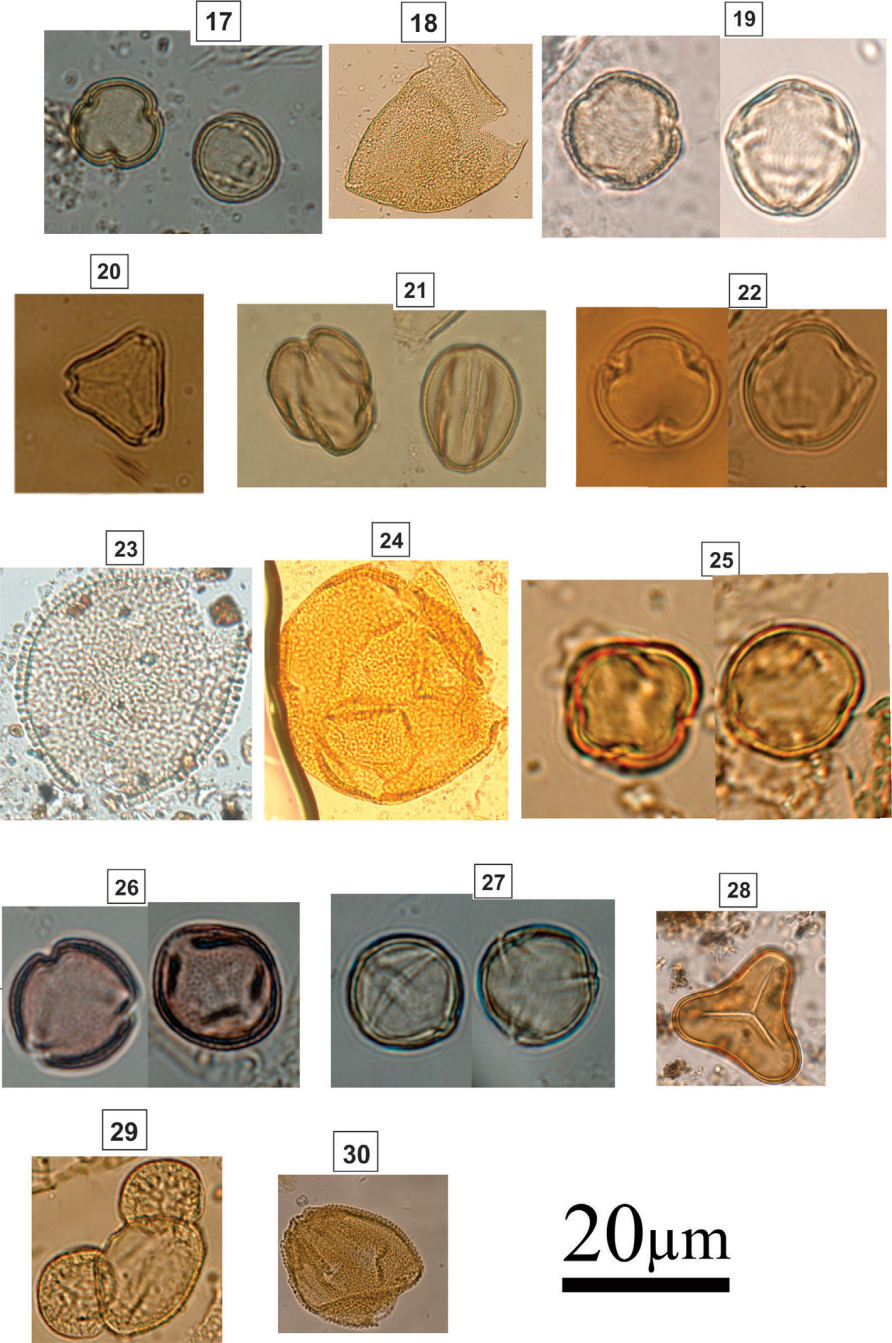
Light micrographs of coastal woodland (restinga) taxa, including: **17.***Pera* sp. (Euphorbiaceae); **18.***Annona* sp. (Anonaceae); **19.***Schinus* sp. (Anacardiaceae); **20.***Myrcia* sp. (Myrtaceae); **21.***Senna* sp. (Fabaceae); **22.***Dalbergia* sp. (Fabaceae); **23.***Cordia* sp. (Boraginaceae); **24.***Opuntia* sp. (Cactaceae); **25.***Myrsine* sp. (Myrsinaceae); **26.***Maytenus* sp. (Celastraceae); **27.***Ricinus* sp. (Euphorbiaceae); **28.***Cyathea* sp. (Cyatheaceae); **29.***Pinus* sp. (Pinaceae); **30.** Passifloraceae

### 2.3 Identification Key (17-30; Fig. 3)

17. ***Pera* sp. (Euphorbiaceae)**

Subspheroidal to prolate; tricolporate; reticulate; circular amb; prolate spheroidals; narrow and long colpus; polar axis: 15–20 µm; equatorial axis: 16–21 µm

18. ***Annona* sp. (Anonaceae) [****2****]**

Oval grain; Monosulcate; reticulate; dispersed in tetrahedral tetrad.

19. ***Schinus* sp. (Anacardiaceae)**

Subspheroidal grain; tricolporate; finely reticulate; colpus length: 18–24 µm; lalongate pore; polar axis: 21–26 µm; equatorial axis: 20–23 µm.

20. ***Myrcia* sp. (Myrtaceae)**

Triangular grain; parasyncolporate; psilate; size: 23–28 µm; colpus length: 18–24 µm; lalongate pore; polar axis: 21–26 µm; equatorial axis: 20–23 µm.

21. ***Senna* sp. (Fabaceae)**

Subspheroidal grain; tricolporate; reticulate; colpus length: 17–23 µm; round pore; polar axis: 27–32 µm; equatorial axis: 21–23 µm.

22. ***Dalbergia* sp. (Fabaceae)**

Subspheroidal grain; tricolporate; rugulate; colpus length: 10–18 µm; lalongate pore; polar axis: 23–29 µm; equatorial axis: 24–32 µm.

23. ***Cordia* sp. (Boraginaceae)**

Suboblate grain; isopolar; radially symmetric; tricolporate; sexine echinate-scabrate; amb circular; grain; polar axis: 25 µm; equatorial axis: 48 µm.

24. ***Opuntia* sp. (Cactaceae)**

Spherical grain, spherical to elliptical; unopened (inaperturado); reticulate; homobrocades; size: 83 µm.

25. ***Myrsine* sp. (Myrsinaceae)**

Spherical grain; isopolar; radially symmetric; stephanocolporate; sexine slightly scabrate; tectated exine; circular amb; polar axis: 20 µm; equatorial axis: 28 µm.

26. ***Maytenus* sp. (Celastraceae)**

Spherical grain; isopolar; bilateral symmetry; circular polar view; subspheroidal equatorial view; tricolporate; long colpi; reticulate sexine; semitectated exine.

27. ***Ricinus* sp. (Euphorbiaceae)**

Suboblate to circular grain; tricolporate; microreticulate; circular to subtriangular amb; colpus width: 0.5–3 µm; lalongate pore; polar axis: 23–30 µm; equatorial axis: 19–24 µm

28. ***Cyathea* sp. (Cyatheaceae)**

Trilete spore; heteropolar; radially symmetry; subtriangular amb; sclerine psilate, radius of the trilete mark (laesura) straight and narrow; size: 48–54 µm.

29. ***Pinus* sp. (Pinaceae) [****3****]**

Bladders with internal reticulum; vesiculate; 34–40 µm.

30. **Passifloraceae**

Spherical grain; sexine reticulate; polar axis: 40.3–43.7 µm; equatorial axis: 39.1–42.2 µm; Woody vines; Herbaceous.

**Fig. 4:**
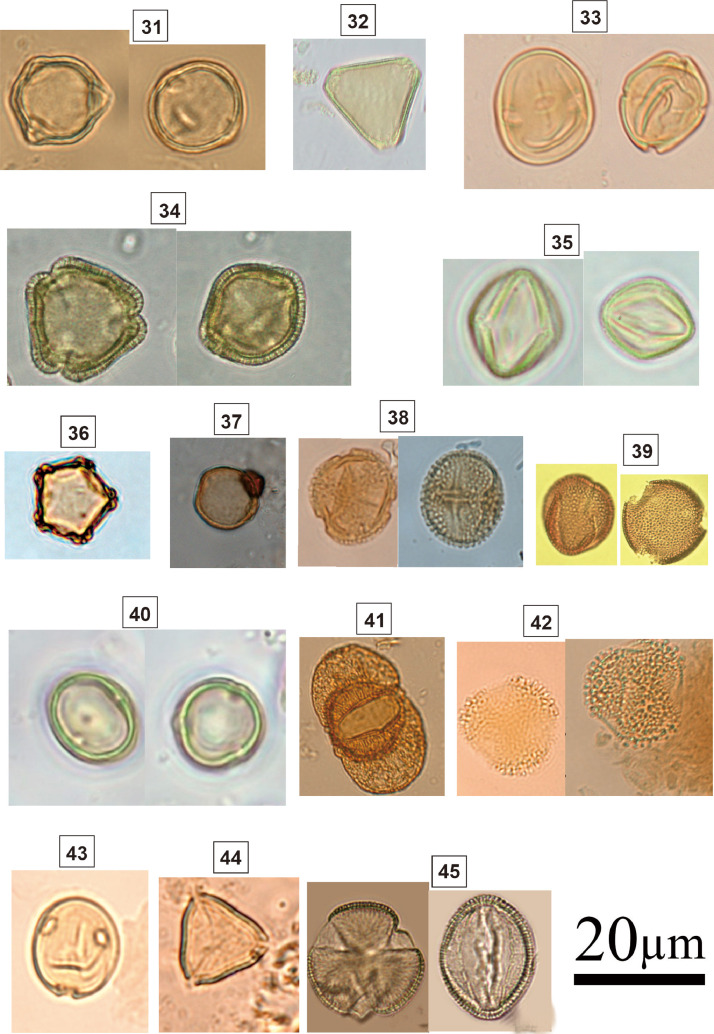
Light micrographs of tropical forest taxa, including: **31.***Dodonaea* sp. (Sapindaceae); **32.***Roupala* sp. (Proteaceae); **33.***Protium brasiliensis* (Burseraceae); **34.***Didymopanax* sp. (Araliaceae); **35.***Slonea* sp. (Elaeocarpaceae); **36.***Alnus* sp. (Betulaceae); **37.***Trema* sp. (Ulmaceae); **38.***Zanthoxylum* sp. (Rutaceae); **39.** Psychotria sp. (Rubiaceae); **40.***Pouteria* sp. (Sapotaceae); **41.***Podocarpus* sp. (Podocarpaceae); **42.***Ilex* sp. (Aquifoliaceae); **43.***Fosteronia* sp. (Apocynaceae); **44.***Eugenia* sp. (Myrtaceae); **45.***Handroanthus* sp. (Bignoniaceae).

### 2.4 Identification key (31-45; Fig. 4)

31. ***Dodonaea* sp. (Sapindaceae)**

Spherical grain; isopolar; radially symmetric; tricolporate; sexine reticulate; triangular to circular amb; elliptical pores; polar axis: 22.8–25.5 µm; equatorial axis: 22.6–26 µm; Shrubs.

32. ***Roupala* sp. (Proteaceae)**

Triangulate grain; isopolar; triporate; straight triangular amb; oblate; microreticulate; thick exine around the pores forming the margin; circular pores; size: 26 µm.

33. ***Protium****brasiliensis***(Burseraceae)**

Spherical grain; isopolar; radially symmetric; tricolporate; sexine psilate; circular amb; prolate; polar axis: 24 µm; equatorial axis: 37 µm.

34. ***Didymopanax* sp. (Araliaceae)**

Suboblate grain; isopolar; radially symmetric; convex-triangular polar view; oblate-spheroidal equatorial view; tricolporate; sexine reticulate; triangular amb; elliptical pores; polar axis: 29 µm; equatorial axis: 34 µm.

35. ***Slonea* sp. (Elaeocarpaceae)**

Olive shaped grain, oblate-spheroidal; isopolar; radially symmetric; tricolporate; sexine slightly psilate appearing as rugulate; circular amb; inconspicuous pores; polar axis: 11 µm; equatorial axis: 13 µm.

36. ***Alnus* sp. (Betulaceae)**

Oblate grain; isopolar; radially symmetric; stephanoporate; sexine psilate slightly scabrate appearing as rugulate; amb circular irregular to pentagonal; size: 21 µm.

37. ***Trema* sp. (Ulmaceae)**

Spherical grain; diporate; microrugulate; round pore; size: 18–21 µm.

38. ***Zanthoxylum* sp. (Rutaceae)**

Subprolate grain; isopolar; radially symmetric; tricolporate; sexine striato-reticulate; circular amb; circular pores; polar axis: 12 µm; equatorial axis: 15 µm.

39. **Psychotria sp. (Rubiaceae)**[4]

Subspheroidal grain; tricolporate; reticulate; colpus length: 19–25 µm; round annulate pore; polar axis: 20–23 µm; equatorial axis: 18–28 µm.

40. ***Pouteria* sp. (Sapotaceae)**

Subprolate grain; isopolar; radially symmetric; tricolporate; sexine psilate; amb circular; elliptical pores; polar axis: 20 µm; equatorial axis: 30 µm.

41. ***Podocarpus* sp. (Podocarpaceae)**

Vesiculate (bisaccate) grain; bilaterally symmetric; sexine reticulate; bisaccate, with air sacs showing fine irregular lines; monoulcerated at the distal pole; medium to large size; Diameter >40 µm.

42. ***Ilex* sp. (Aquifoliaceae)**

Subspheroidal grain; tricolporate; clavate; colpus length: 15–19 µm; lalongate pore; polar axis: 26–29 µm; equatorial axis: 24–28 µm.

43. ***Fosteronia* sp. (Apocynaceae)**

Suboblate to circular grain; zonaporate; circular amb; sexine psilate; polar axis: 28–34 µm; equatorial axis: 32–37 µm; Tree.

44. ***Eugenia* sp. (Myrtaceae)**

Triangular grain; parasyncolporate; psilate; size: 23–28 µm.

45. ***Handroanthus* sp. (Bignoniaceae)**

Spheroidal oblate grain; isopolar; radially symmetric; tricolpate; sexine scabrate; elliptical pores; size: 58–60 µm.

**Fig. 5:**
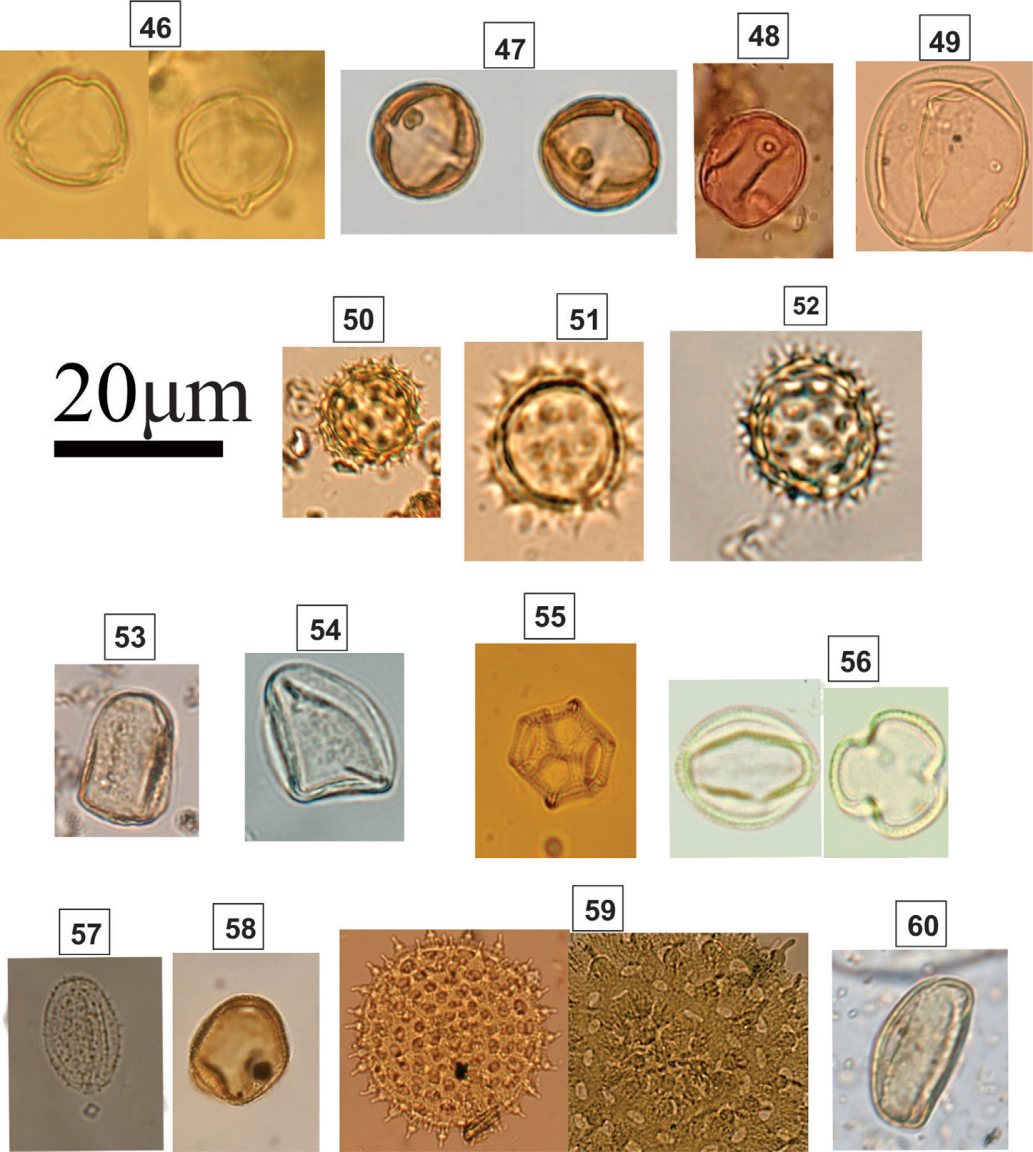
Light micrographs of herbaceous taxa, including **46.***Solanum* sp. 1 (Solanaceae); **47.***Solanum* sp. 2 (Solanaceae); **48.** Poaceae sp. 1; **49.** Poaceae sp. 2; **50.** Asteraceae sp. 1; **51.** Asteraceae sp. 2; **52.** Asteraceae sp. 3; **53.***Cyperus* sp 1. (Cyperaceae); **54.***Cyperus* sp. 2 (Cyperaceae); **55.***Alternanthera* sp. (Amaranthaceae); **56.***Scoparia* sp. (Plantaginaceae); **57.***Smilax* sp. (Smilacaceae); **58.***Typha* sp. (Typhaceae); **59.** Ipomoea (Convolvulaceae); **60.***Eichhornia* sp. (Ponteridadeae)

### 2.5 Identification key (46-60; Fig. 5)

**46&47*. Solanum* sp. (Solanaceae) [** **3** **]**

Subspheroidal to prolate grain; tricolporate–syncolporate; colpus length: 15–22 µm; psilate; lalongate pore; polar axis: 17–26 µm; equatorial axis: 15–25 µm.

**48&49. Poaceae**

Spherical grain; monoporate; scabrate; round annulate pore; polar diameter: 3–4 µm; size: 30–53 µm.

**50-52. Asteraceae**

Subspheroidal grain; tri–tetracolporate – syncolporate; echinate; colpus length: 2–3 µm; pore obscured by sculpture; polar axis: 15–25 µm; equatorial axis: 16–26 µm.

**53&54. *Cyperus* sp. (Cyperaceae)**

Rounded triangular grain; Ulcerate; scabrate; ulcerate; size: 20–28 µm.

**55. *Alternanthera* sp. (**Amaranthaceae**)**

Inaperaturate; lophate; size: 18–25 µm.

**56. *Scoparia* sp. (Plantaginaceae)**

Spheroidal grain; isopolar; radially symmetric; tricolporate; sexine microreticulate; size: 10–25 µm.

**57. *Smilax* sp. (Smilacaceae)**

Monad grains; inaperaturate; spheric; scabrate; size: 16–19 µm.

**58. *Typha* sp. (Typhaceae)**

Spherical grain; tetrad; Monoulcerate; reticulate; individual size: 20–25 µm.

**59. *Ipomoea* (Convolvulaceae)**

Spherical grain; periporate; round pores.

**60. *Eichhornia* sp. (Ponteridadeae)**

Oval grain; monosulcate; microverrucate; size: 30–50 µm.

## 3 Experimental Design, Materials and Methods

The sediment archive (core SF-5; 100 cm) that hosts all the pollen taxa described in this dataset was retrieved in September 2015 using a Russian Peat Borer from an estuarine mangrove swamp adjacent to the upstream of Palmital River's main channel (26°06′48.10′′ S, 48°47′17.00′′ W). Fifty samples were taken a 2 cm interval throughout the core for palynological analysis. All samples were processed following an optimized preparation method that eliminates the organic and clastic materials and concentrate the pollen grain [5]. First, carbonate materials in the extracted sediment samples were eliminated with hydrochloric acid. Second, we removed organic materials from the samples with potassium hydroxide. Last but not the least, clastic materials ranging from coarse sand (> 200 µm) to clay (< 10 µm) were removed with sieving and hydrofluoric acid. All pollen samples were identified via an Olympus Light Microscope, and the light micrograph of all pollen taxa described in this study was taken with a LC35 camera (3.5-megapixel CMOS sensor) in conjunction with ZEN Blue 2012 imaging software under 1000x magnification. TILIA and TILIAGRAF software were used for calculation and plotting the pollen diagram [6]. CONISS was used for cluster analysis of the pollen taxa [7].

## Ethics Statements

This study does not involve any human subjects or animal experiments.

## CRediT authorship contribution statement

**Erika Rodrigues:** Formal analysis. **Marcelo Cancela Lisboa Cohen:** Resources. **Beatriz L Figueiredo:** Visualization. **Kam-biu Liu:** Writing – review & editing. **Qiang Yao:** Writing – original draft.

## Declaration of Competing Interest

The authors declare that they have no known competing financial interests or personal relationships that could have appeared to influence the work reported in this paper.

## Data Availability

Pollen dataset from Santa Catarina, south Brazil (Original data) (Mendeley Data).
